# Morphological and Molecular Descriptors of the Developmental Cycle of *Babesia divergens* Parasites in Human Erythrocytes

**DOI:** 10.1371/journal.pntd.0003711

**Published:** 2015-05-08

**Authors:** Ingrid Rossouw, Christine Maritz-Olivier, Jandeli Niemand, Riette van Biljon, Annel Smit, Nicholas A. Olivier, Lyn-Marie Birkholtz

**Affiliations:** 1 Department of Genetics, University of Pretoria, Pretoria, South Africa; 2 Department of Biochemistry, Centre for Sustainable Malaria Control, University of Pretoria, Pretoria, South Africa; 3 Department of Plant Sciences, African Centre for Gene Technologies (ACGT) Microarray facility, University of Pretoria, Pretoria, South Africa; University of Tennessee, UNITED STATES

## Abstract

Human babesiosis, especially caused by the cattle derived *Babesia divergens* parasite, is on the increase, resulting in renewed attentiveness to this potentially life threatening emerging zoonotic disease. The molecular mechanisms underlying the pathophysiology and intra-erythrocytic development of these parasites are poorly understood. This impedes concerted efforts aimed at the discovery of novel anti-babesiacidal agents. By applying sensitive cell biological and molecular functional genomics tools, we describe the intra-erythrocytic development cycle of *B*. *divergens* parasites from immature, mono-nucleated ring forms to bi-nucleated paired piriforms and ultimately multi-nucleated tetrads that characterizes zoonotic *Babesia* spp. This is further correlated for the first time to nuclear content increases during intra-erythrocytic development progression, providing insight into the part of the life cycle that occurs during human infection. High-content temporal evaluation elucidated the contribution of the different stages to life cycle progression. Moreover, molecular descriptors indicate that *B*. *divergens* parasites employ physiological adaptation to *in vitro* cultivation. Additionally, differential expression is observed as the parasite equilibrates its developmental stages during its life cycle. Together, this information provides the first temporal evaluation of the functional transcriptome of *B*. *divergens* parasites, information that could be useful in identifying biological processes essential to parasite survival for future anti-babesiacidal discoveries.

## Author Summary

Vector-borne parasitic diseases are still the major cause of morbidity and mortality in both humans as animals. Some of these parasites have been well studied, including the malaria parasite, *Plasmodium falciparum*, since these cause major fatalities in humans. However, other parasites like *Babesia divergens*, who closely resemble malaria parasites, are currently becoming a major concern since these are zoonotically transmitted to humans from their natural hosts (e.g. cattle) by ticks. Their rising levels indicate the possibility of human medical emergencies occurring. Unfortunately, basic biological processes in these parasites are poorly understood due to their neglected status. In this manuscript, we describe for the first time high-content analysis of the development of *B*. *divergens* parasites *in vitro* over time. We applied cell biological and advanced functional molecular strategies to also provide the first descriptors of the parasite’s transcriptome during life cycle development. This information is unique and to not only dramatically expand our information base on *Babesia* biology, but also provide information that can be exploited for future drug discovery endeavors.

## Introduction

Human babesiosis is a rapidly emerging, zoonotic, infectious disease causing potentially life-threatening malaria-like symptoms in humans. It is caused by intra-erythrocytic protozoan parasites of the genus *Babesia* [1] and it is transmitted to humans *via* an ixodid tick vector or through a blood transfusion from asymptomatic carriers [2]. Bovine babesiosis is well regarded as one of the most important diseases of livestock, especially in the tropical and sub-tropical regions of the world [3]. However, human babesiosis disease prevalence has escalated over the past 50 years from a few isolated cases to global endemic areas now being recognized [4,5]. In Europe, cattle associated *B*. *divergens* is the most common causative agent of human babesiosis, especially throughout regions with extensive cattle industries, as the distribution geographically correlates with both pathogen infected host species and tick-vector infested regions, allowing for zoonotic transmission potential [6].

Disease burden outside North America and Europe is currently poorly described but considering the worldwide distribution of *Babesia* parasites, improved surveillance is required. Since the symptoms of human babesiosis resemble that of malaria and diagnosis is predominantly reliant on microscopic evaluation of blood smears, this disease may be misdiagnosed as a malaria infection, especially in areas of co-endemicity. Early disease detection, diagnosis and treatment with effective anti-babesiacidal compounds are therefore vital for both human and animal health [7]. In humans, *Babesia* parasites can be cleared by anti-malarials including atovaquone (with azithromycin) or quinine (plus clindamycin) but highly immuno-compromised individuals respond poorly to these treatments. As early reports of resistance against these combinations have been noted in the past few years, the need for alternative treatments is evident [8,9].

Against this background of potential zoonotic human babesiosis medical emergencies, it is quite surprising that our understanding of the basic biological processes underlying *Babesia* pathophysiology is still poorly understood, even with the recent application of genetic manipulation for transfection of *Babesia* parasites as well as the sequencing of the *B*. *bovis* genome [10,11]. Particularly intriguing is the fact that the precise progression and duration of the intra-erythrocytic, asexual developmental cycle (IDC) has not been clarified. During its IDC, *Babesia* parasites undergo asexual replication by binary fission (budding) of trophozoites to form 2–4 merozoites [12]. Each merozoite is thought to undergo a single cycle of division and then escape *via* cell lysis to re-infect new erythrocytes [13]. This establishes a perpetual, asynchronous asexual parasitic growth cycle, which is thought to last approximately 8 hours [14] and encompasses several developmental stages all present at the same point in time within the hosts’ bloodstream [15]. However, the description of the IDC and its different stages are fraught with uncertainties: historically different stages were described only based on light microscopy; little attention has been paid to their sequence of development and descriptions of the various *Babesia* parasitic *in vitro* stages do not share a consensus in literature and display considerable morphological pleiomorphism. Moreover, fundamental biological questions remain unanswered, particularly concerning the molecular descriptors governing the IDC of *Babesia* parasites.

In this study, the *in vitro* IDC of the human pathogen *B*. *divergens* was comprehensively evaluated by employing various high-content cell biological and molecular strategies as has been previously applied to the more widely studied but related hemoprotozoan malaria parasite, *Plasmodium falciparum*. The study particularly focused on *B*. *divergens* as human pathogen and model organism for *Babesia* since it is amenable to *in vitro* cultivation. This is to our knowledge the first quantitative description and temporal evaluation of intra-erythrocytic *B*. *divergens* development and enabled clear characterization of the stage-specific development, based on nuclear proliferation in these parasites. The information is novel not just from a biological perspective, but will also be essential in future prioritization of anti-babesiacidal compounds.

## Results

### Evaluation of *B*. *divergens in vitro* blood stage development

#### Morphological discrimination of life cycle stages

**Fig 1:**
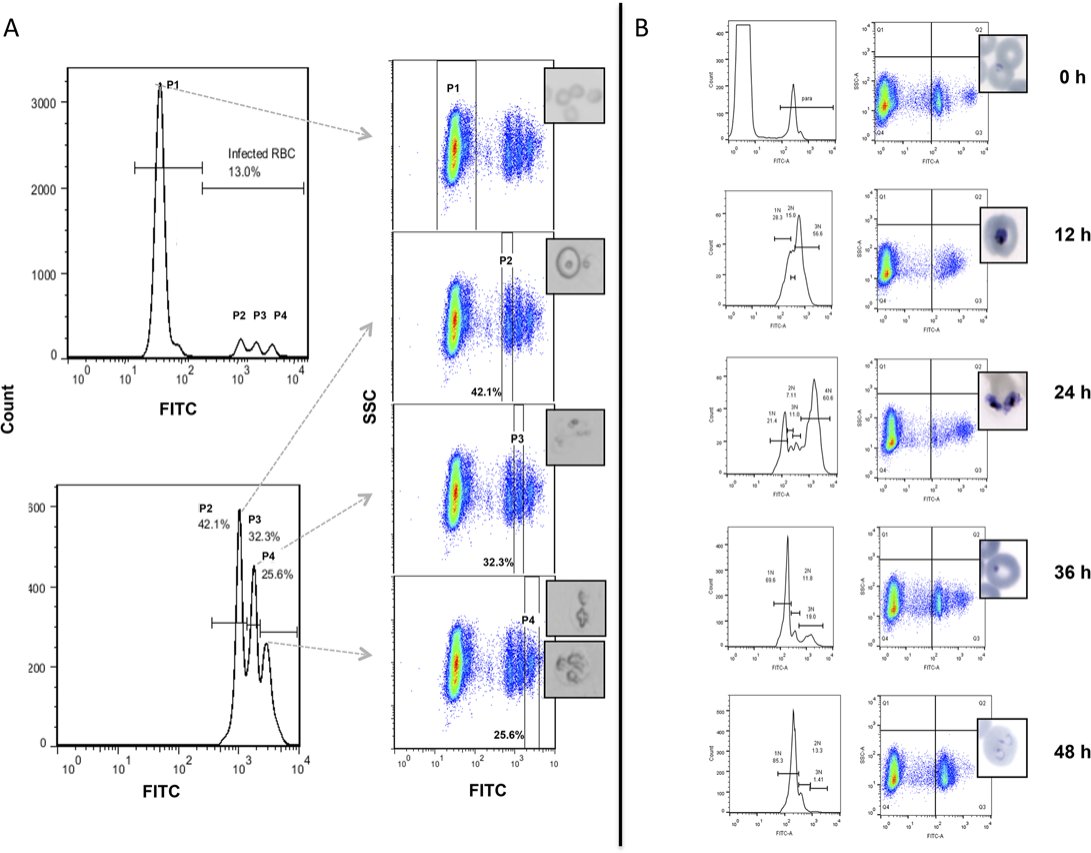
Morphological discrimination of *B*. *divergens in vitro* blood stage development. (A) Flow cytometric developmental stage differentiation, sorting and isolation of intra-erythrocytic *B*. *divergens* parasites *via* SYBR Green I staining of DNA tracked in the FITC channel. Histogram panels indicate the gated populations as either uninfected (P1) or infected erythrocytes (P2; P3; P3), as well as the percentage infected erythrocytes (parasitemia). Light microscopy images illustrate the individually sorted and isolated populations as either uninfected (P1) or infected (P2-P3) erythrocytes. Enriched parasite formations include ring (P2), paired piriform (P3) and tetrad and/or multiple infections (P4). (B) Flow cytometric evaluation of intra-erythrocytic *P*. *falciparum* development over 48 hours as a control system to prove the ability to distinguish between different parasite populations in an intra-erythrocytic developmental cycle. Synchronized *P*. *falciparum* parasites could be distinguished as either ring forms containing single nuclei, 1N at 0–12 h post invasion, mature trophozoites (2-4N nuclear content) and schizonts (>4N nuclear content) (24 h post invasion) resulting in daughter merozoites (1N nuclear content) re-infecting new erythrocytes at 36–48 hours post invasion.

We developed an optimal technique to detect intra-erythrocytic B. divergens parasites using fluorescent tracking through flow cytometry (S1 Text). This flow cytometric assay showed a strong linear correlation with light microscopy, enabling quantitative analyses of these parasites (S1 Fig). The fluorescent detection of intra-erythrocytic *B*. *divergens* parasites enabled for the first time further interrogation of the population diversity within unsynchronized *B*. *divergens* cultures. From the data presented in S1 Fig, distinct infected erythrocyte populations were observed. We subsequently questioned if these could be correlated to specific parasitic developmental forms by subsequent gating, sorting and isolation of individual cells and cell populations (Fig 1A). By gating for the intra-erythrocytic *B*. *divergens* populations, three distinct sub-populations could be independently sorted, microscopically evaluated and classified [15,16]. The majority (± 67%) of parasitized erythrocytes from the second population (P2) contained ring formations while the majority associated with the third population (± 58%; P3) and fourth (± 50%; P4) populations contained paired piriforms and tetrads, respectively (Fig 1A). The various infected populations were not present in equally distributed proportions. Ring forms, paired piriforms and tetrads represented 42%, 32% and 26% of the total infected erythrocytes, respectively. Similarly, the more synchronous development of intra-erythrocytic *P*. *falciparum* parasites could be evaluated (Fig 1B). Immature ring-stage *P*. *falciparum* parasites were distinctly visible (mono-nucleated forms, first panel at 0 h and developed through metabolizing trophozoite forms to the multi-nucleated schizonts within 24 h (61% population distribution, Fig 1B). The newly formed daughter merozoites resulting from schizogony (70% population distribution, 36 h) were able to re-infect new erythrocytes, initiating a subsequent development cycle since ring-stage parasites were detected at 48 h.

#### Nuclear content associated with *B*. *divergens* parasites’ life cycle compartments

**Fig 2:**
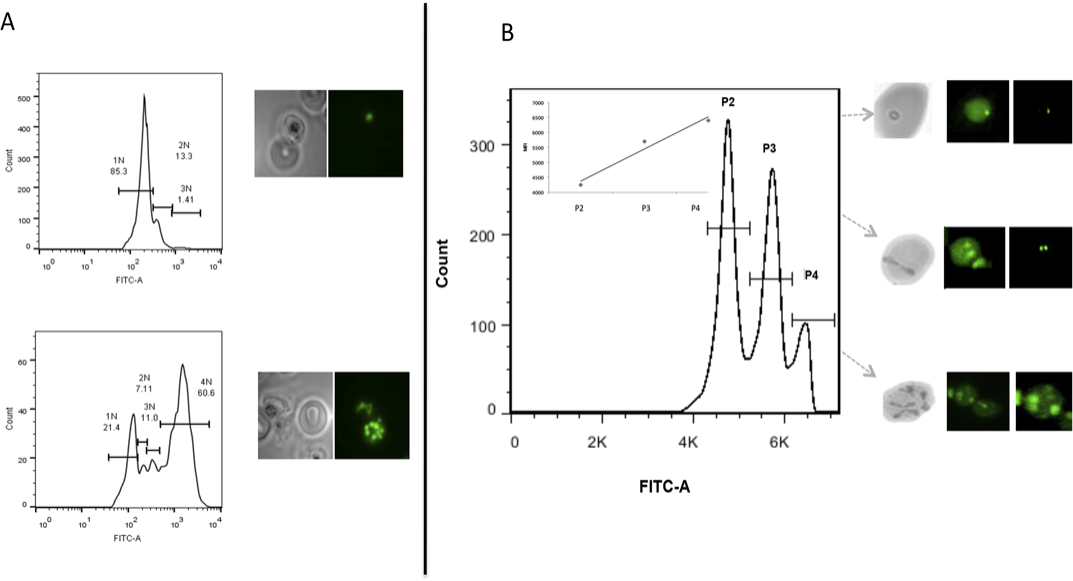
Correlation of nuclear content to IDC compartments (stages) for *B*. *divergens* and *P*. *falciparum* parasites. Flow cytometric evaluation of different parasite populations was performed as for Fig 2 and parasite populations additionally analyzed with fluorescent microscopy (1:100 SYBR Green I stained, 30 min dark at room temperature and evaluated with green filter). (A) Immature, ring-stage *P*. *falciparum* parasites containing a single nucleus with 1N DNA content (top) and multi-nucleated schizont forms (at >5 distinct nuclei with >5N DNA content; bottom). (B) *B*. *divergens* population discrimination based on a linear scale with corresponding median fluorescent intensities (MFI) values. MFI values for each isolated population (indicated by grey arrows) ranged between P2 and P3 (4520–5700 units) and between P3 and P4 (5200–6400 units) and increased linearly (R^2^ value of 0.96) between the three observed, infected populations. Fluorescent microscopy images visualized DNA nuclear content for each infected *B*. *divergens* erythrocyte population as (P2) single ring formation with a single parasitic nucleus (1N), (P3) paired piriform with two nuclei (2N) and (P4) tetrad and/or multiple infections with two or more nuclei (>2N).

The ability to discriminate between distinct populations of intra-erythrocytic life stages of *B*. *divergens* parasites is highly informative considering that *B*. *divergens* cultures cannot be synchronized *in vitro* (compared to for instance *P*. *falciparum* cultures). Since SYBR Green I has shown to be effective as a stoichiometric marker of DNA content in malaria parasites, it can be used to further evaluate the progression of parasite development during the intra-erythrocytic developmental cycle, based on changes in nuclear content [17]. In Fig 2A, a clear distinction is observed between immature, ring-stage *P*. *falciparum* parasites (containing a single nucleus with 1N nuclear content) compared to mature, multi-nucleated schizont forms of the parasite where more than 5 distinct nuclei (>5N nuclear content) could be distinguished. Comparatively, fluorescent analysis of the nuclear content of the mixed intra-erythrocytic *B*. *divergens* parasite cultures revealed a clear correlation between DNA fluorescence and nuclear bodies. Median fluorescence intensities (MFI) increased linearly (R^2^ = 0.96) between the three observed, infected populations. The MFI values increased from ring to paired piriform populations of *B*. *divergens* parasites (P2 to P3 increased from 4520–5700 units) as well as between paired piriforms to tetrads (P3 and P4 from 5200–6400 units). Fluorescent microscopy confirmed the observed morphological findings associated with stage-specific division of each infected population and subsequently indicated nuclear content (Fig 2B). As such, ring stage parasites contained single, clear nuclei (P2). Distinct V-shaped nuclei characterized the bi-nucleated paired piriforms (P3) and the tetrads (P4) were distinguished by at least 3–4 multiple but distinct nuclei. However, the presence of multiple infections of mono-nucleated parasites in both paired piriform and more specifically tetrad populations cannot be ruled out.

### Temporal evaluation of *B*. *divergens* parasites’ life cycle progression

**Fig 3:**
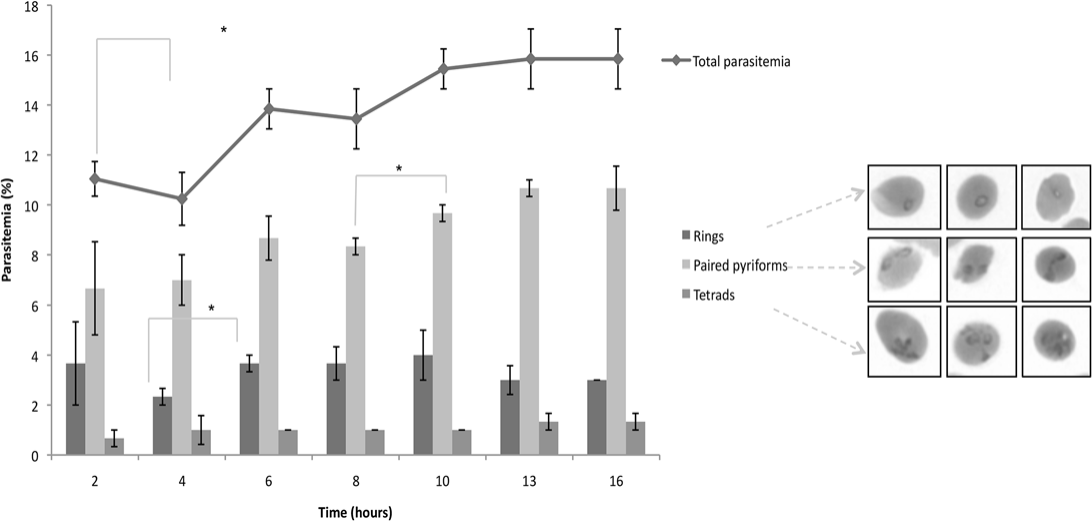
Temporal evaluation of *B*. *divergens* progression over two parasitic life cycles. An established *B*. *divergens in vitro* culture was re-inoculated into fresh media (at time 0) and temporal evaluation of the life cycle stage distribution performed every 2 hours over a total 16-hour period. Giemsa stained microscopy was performed and parasite stages classified morphologically as ring, paired piriforms and tetrads infections according to [16]. Total parasitemia is indicated per time point analyzed (line graph) and compared to the contribution of each life cycle stage (% of total parasitemia) at each time point. Results are the mean of three independent experiments, performed in triplicate (± S.E.). Significance is indicated at *P*<0.05 (*) as determined with a unpaired Student-t test.

Based on the findings presented here that correlate the linear increase of DNA content to its associated nuclear content and morphological classification, the progression of intra-erythrocytic *B*. *divergens* parasites in its development from one IDC stage to another could be proposed. Temporal evaluation of *in vitro* proliferation for each developmental form (ring, paired piriforms, tetrads and/or multiple infections) was performed over a 16-hour period. Parasitemia steadily increased to 15% over the 16-hour period monitored in the asynchronous culture (Fig 3). However, between 4–6 hours of development, the ring formation population doubled (from a 2% to a 4% contribution to parasitemia, *P*<0.05, n = 3) (Fig 3). This was additionally associated with an increase in paired piriforms (2% increase in contribution to total parasitemia for paired piriforms). Similar observations were made between 8–10 hours of development, where a significant increase in paired piriforms was again observed (8% to 10%, *P*<0.05, n = 3) (Fig 3). Overall, the population distribution in the mixed culture remained predominantly paired piriforms (9.04 ± 1.03% contribution to total parasitemia that ranged from 10.3–15.3%) followed by ring formations (3.33 ± 0.68% contribution to total parasitemia) (Fig 3). The tetrad (or multiple infection) population is the most stable throughout the temporal evaluation with no significant variation in population size (average 1.08 ± 0.28% contribution to total parasitemia maintained throughout).

The increase in ring and piriform populations between 4–6 hours of development additionally contributed to a 3% increase in total parasitemia (from 10 to 13% total parasitemia) and again a 2% increase in parasitemia between 8–10 hours (from 13 to 15% total parasitemia). This is indicative of life cycle progression through re-infection of erythrocytes (contributing to new rings formed) and parasite developmental maturation (contributing to new paired piriforms). If significant increases in the ring populations are taken as an indicator of merozoite invasions of erythrocytes and initiation of new development cycles, it appears therefore that the *in vitro* development of *B*. *divergens* is typified by a 4 hour progression window and that, after two parasitic life cycles and under the culture conditions employed, equilibrium could be established and maintained. As such, a multiplication index of 3-fold was subsequently observed for continued *B*. *divergens in vitro* development.

### Molecular descriptors characterizing the life cycle of *B*. *divergens* parasites

**Fig 4:**
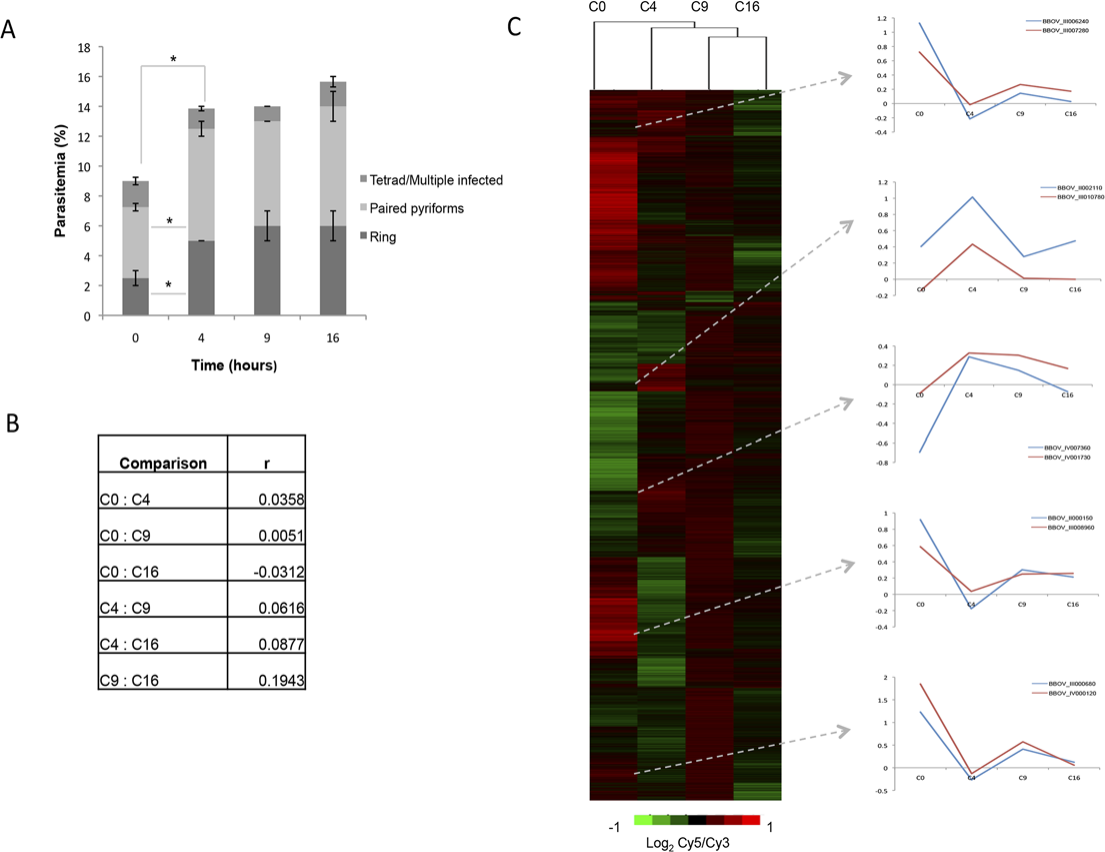
Molecular descriptors characterizing the IDC of *B*. *divergens* parasites. (A) Morphological evaluation of *B*. *divergens* life cycle progression from a newly initiated culture over a total of 16 hours, indicating the distribution of life cycle stages to total parasitemia. (B) Pearson correlations of the mRNA abundance levels of *B*. *divergens* parasites in an initiate culture compared to parasites that adapted to culture for 4, 9 and 16 hours, respectively. (C) Cy5-labelled cDNA from *B*. *divergens* parasites across four different times (0, 4, 9, 16 hours post-inoculation) were hybridized to Cy3-labelled cDNA from a reference pool spanning all samples. Transcripts were clustered according to expression values (M-values, log_2_ Cy5/Cy3) of the initiate culture, with the expression profiles of representative genes indicated. On the heat map, scale (log_2_ Cy5/Cy3) is from 1 to -1. Transcripts were classified based on their dCAS annotation and COG classification compared to *B*. *bovis* homologs.

There are currently no data describing the molecular events associated with *B*. *divergens* intra-erythrocytic development, information that is essential to understanding the nature of stage-specific progression of this parasite’s IDC. Since clear contributions of morphologically distinct life cycle compartments were observed to contribute to the IDC progression of *B*. *divergens* parasites, we set out to describe the global transcriptome of these parasites through its IDC as an indicator of the physiological processes involved. Transcriptome analysis (mRNA abundance determination) was subsequently conducted on asynchronous, newly initiated cultures with a custom designed DNA microarray containing 15744 target features covering 97% of the *B*. *bovis* genome (3703 independent ORFs) and using a reference pool design strategy (Fig 4).

*B*. *divergens* parasites seem to adapt to culturing under unlimited growth conditions (4% increase in parasitemia, *P*<0.05, n = 3) (Fig 4A). Again, this adaptation was resultant of a significant increase in both ring and paired piriform parasites (from 2.5 to 5% and 4.75 to 7.5%, respectively, *P*<0.05, n = 3); after this the parasite population distribution equilibrated. These observations were further evident from correlation data, indicating that the transcriptome of equilibrated parasites showed the best correlation (r = 0.19) between parasites that have been in culture for 9 and 16 hours (C9 vs. C16, Fig 4B). Comparatively, initiate cultures showed complete disconnect from established cultures (C0 vs. C16 anti-correlated at -0.03). This clearly indicates a physiological adaptation event underscored by a predominant transcriptional repression in the initiate culture (931 undefined transcripts, 387 transcripts with increased abundance, compared to 2385 repressed transcripts). Differential expression between the initiate culture and the parasites 4 hours after inoculation resulted in 164 transcripts significantly affected (82 decrease and increase in abundance, respectively) (S1 Table). Of these, only 50 could be annotated using the dCAS annotation system [18] based on each transcript’s COG classification (*E-*value cut-off of less than 1x10^-4^) (Table 1). The processes mostly affected include transcription (26%), translation (8%), protein turnover (21%), cellular (30%) and metabolic (11%) processes, and stress defense mechanisms (2%). All activated transcripts may be associated with the changes in parasite population distributions (increases in ring and paired piriforms). Clustering of transcripts based on co-expression profiles indicated the expected similarity in expression profiles across the transcriptome (Fig 4C). Moreover, transcripts in some of these co-expressed clusters additionally showed clear chromosomal synteny (e.g. BBOV_III006240 and BBOV_III007280 on chromosome III; BBOV_IV007380 and BBOV_IV001730 on chromosome IV) implying transcriptional level regulation for these transcripts.

**Table 1:** Biological functions of a subset of transcripts associated with differential abundance in *B*. *divergens* parasites upon culture adaptation.

Transcript ID (PiroplasmaDB)	Annotation	logFC^*a*^
**Translation associated**		
BBOV_III000190	ATP-dependent RNA helicase	-4.375312774
BBOV_IV001200	ATP-dependent RNA helicase	-2.174634998
BBOV_I000180	CCAAT-binding factor	-1.834245335
BBOV_III004170	Exosomal 3'-5' exoribonuclease complex	1.712442768
BBOV_II002690	Global transcriptional regulator	-2.346777377
BBOV_II005600	mRNA splicing factor	1.590307877
BBOV_IV006920	mRNA splicing factor	2.007870414
BBOV_I002600	RNA polymerase II	2.338999862
BBOV_III000960	RNA polymerase III	-2.020414056
BBOV_III011610	RNA pseudouridylate synthases	2.116373472
BBOV_I004230	Small nuclear ribonucleoprotein (snRNP)	1.121027401
BBOV_III008510	Spliceosomal protein FBP21	-2.346219667
BBOV_IV004470	U4/U6-associated splicing factor PRP4	-1.58664859
BBOV_III005690	Uncharacterized mRNA-associated protein	2.25214499
**Translation associated**		
BBOV_III000980	60S ribosomal protein L7	1.059907286
BBOV_IV001690	Aspartyl-tRNA synthetase	1.466182198
BBOV_IV006160	Mitochondrial polypeptide chain release factor	-2.421480872
BBOV_IV007450	Mitochondrial/chloroplast ribosomal protein L2	2.098909746
**Protein turnover**		
BBOV_II002330	20S proteasome, regulatory subunit	1.58165857
BBOV_IV004490	Chaperone-dependent E3 ubiquitin protein ligase	1.097695749
BBOV_II005540	Cysteine protease required for autophagy	-1.679993311
BBOV_III000400	Para-hydroxybenzoate-polyprenyl transferase	1.301434275
BBOV_II007270	Predicted small molecule transporter	1.030631755
BBOV_IV006860	SCF ubiquitin ligase	-4.352294731
BBOV_IV004330	Serine protease	-1.680191261
BBOV_III009930	Ubiquinol cytochrome c reductase	-1.800386366
BBOV_IV001730	Ubiquitin carboxyl-terminal hydrolase	-2.434145021
**Cellular process**		
BBOV_II004950	Adenylosuccinate synthase	-2.103832513
BBOV_II005700	Ca2+ transporting ATPase	-2.00987223
BBOV_IV008310	Ethanolamine kinase	-2.348186354
BBOV_IV005830	F0F1-type ATP synthase	2.061846648
BBOV_IV010920	GTPase Ran/TC4/GSP1	0.966482698
BBOV_III008970	Membrane coat complex retromer	-3.015783812
BBOV_IV005570	Metallopeptidase	-2.564704469
BBOV_IV003180	Predicted transporter	1.338554072
BBOV_IV000850	Protein containing U1-type Zn-finger	2.078642004
BBOV_I001560	Protein kinase	-1.208185035
BBOV_I001930	Putative arsenite-translocating ATPase	-1.744110089
BBOV_III007370	Serine/threonine protein kinase	-2.604703317
BBOV_III006690	Vesicle coat complex AP-1/AP-2/AP-4	-2.228269371
BBOV_I001880	Vesicle coat complex AP-2	-1.607418322
BBOV_II002960	Vesicle coat complex COPI	-1.541188504
BBOV_IV000740	Vesicle coat complex COPII	1.202748703
**Metabolic process**		
BBOV_IV000490	Acyl-CoA-binding protein	-2.367008881
BBOV_II006920	Citrate synthase	-2.554668131
BBOV_IV007190	Dihydrolipoamide dehydrogenase	2.002751088
BBOV_III009670	Dihydroorotase	-2.140622832
BBOV_IV001010	Fe-S oxidoreductase	1.445685397
BBOV_IV007210	Succinate dehydrogenase	2.924498163
**Stress defence**		
BBOV_II006560	Molecular chaperone (DnaJ superfamily)	2.040609594

^a^ Average fold change

**Fig 5:**
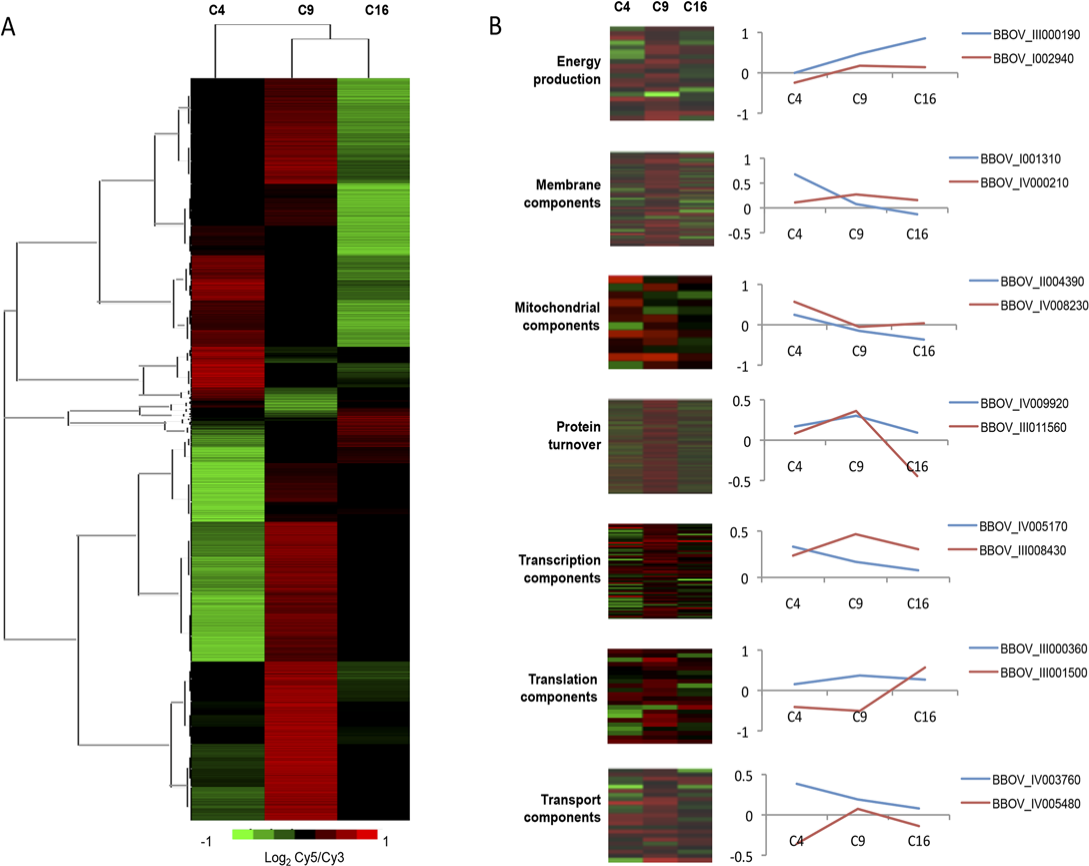
Biological processes involved in *B*. *divergens* parasites’ intra-erythrocytic development. Transcripts from adapted cultures at 4, 9 and 16 hours were analyzed based on their *M-*values (log_2_ Cy5/Cy3) and visually inspected to identify an alternative parasitic expression pattern (A). Transcripts were clustered based on their shared expression profiles in the 4-hour culture (C4). Functional classification was performed based on GO-cellular compartment, molecular function and biological process domains (B).

Further temporal evaluation of the *B*. *divergens* transcriptional landscape during its IDC indicated correlation across the transcriptional landscape (Fig 5). However, clear transcriptional activation to a permissive state was observed as the parasites progressed in their life cycle, particularly evident for parasites in culture for at least 9 hours (e.g. in C4, 45% of the transcripts showed increased abundance; this increased to 60% at C9) (Fig 5A). However, after 16 hours in cultivation, the transcriptome seems more unbiased and show a more equal distribution of abundances in transcripts with an inclination towards transcriptional repression. This may be correlated to a slight increase in the paired piriform population in the morphological profiles observed between parasites in culture for 9 or 16 hours. Further comparison of the transcriptomes of the parasite populations indicated the presence of differential expression patterns across the 4–16 hour evaluations. To distinguish only processes associated with IDC progression and minimize adaptation responses as a result of culture initiation (time 0–4 h), transcriptional profiles were evaluated between parasites in culture for 4, 9 and 16 hours, respectively (Fig 5B). Functional annotation of transcripts associated with the alternative expression patterns identified several transcripts associated with normal biological, cellular and functional pathways throughout the investigated period. This included 11 major functional categories (catalytic activity 16%; energy production and conversion 5%; lipid transport metabolism and synthesis 2%; membrane protein components 6%; mitochondrial components 3%; proteolysis 13%; ribosomal components 17%; transcription and translation 25%; transport 6%, and variant surface antigen expression 9%), which were present across all clusters and time points. However, certain biological processes proved more variable over the temporal evaluation, particularly protein turnover, transcription and translation with increased levels of activity at 9 hours in culture. Comparatively, energy production is, as expected, maintained throughout the temporal evaluation.

## Discussion

Our current understanding of the intra-erythrocytic developmental cycle of *B*. *divergens* has been clouded by imprecise and conflicting classifications, mostly due to historical analyses relying for the most part only on morphological microscopic observations. For instance, discrepancies in morphological evaluation of different life cycle forms are reported, where trophozoites are sometimes referred to as merozoites [12]. Additionally, one of the major challenges with studying the stage-specific development of *B*. *divergens* intra-erythrocytic development is the inability to synchronize these parasites *in vitro* to a single stage. A well-developed synchronization strategy induced by either sorbitol or mannitol is widely applied in *P*. *falciparum* research and is mainly associated with changes in membrane permeability and buoyant density of erythrocytes parasitized by this organism [19]. However, these techniques were evaluated and found to be ineffective against *B*. *divergens in vitro*, resulting in parasite death within 24 hours.

The data obtained by combining advanced cell biological and molecular strategies allow for the first time objective and clear evaluation of the chronology and stage-specificity of intra-erythrocytic development of *B*. *divergens* parasites. These are sensitive and quantitative and allow various developmental stages to be distinguished. Flow cytometry, light and fluorescent microscopy allowed for the accurate detection of intra-erythrocytic *B*. *divergens* parasites as well as determination of dynamic proliferation, developmental stage assessment and isolation of asynchronous *B*. *divergens* parasites, based on morphology as well as nuclear content.

Asynchronous, independent nuclear division occurs during intra-erythrocytic *P*. *falciparum* development, where daughter merozoites follow a non-geometric expansion and parasitic multiplication consequently deviates from what is expected from equal numbers of binary divisions [20]. Similar findings were observed with light microscopy and flow cytometry for the asynchronous *in vitro B*. *divergens* cultures in this study and enabled the isolation of specific developmental stages over a 16-hour period. The increase in MFI values observed between the three infected populations corresponds to the fluorescent microscopy images, which ultimately indicate an increase in DNA nuclear content from one developmental stage to the next. Based on the DNA measurements, which underlie the morphological findings of the present study, asynchronous *in vitro Babesia* dynamics was further evaluated. With primary parasitology classifications in mind, intra-cellular and actively metabolizing parasites are usually classified as trophozoites, which divide asexually (merogony) with the resultant formation of daughter merozoites. Erythrocytes infected with a ring formation, contain a single parasitic nucleus (1N) and erythrocytes infected with either paired piriforms, tetrads or multiple infected erythrocytes, contain two or more nuclei (2N or >2N). These findings correlate nuclear content to a particular isolated cell population (based on morphology); previously unclear for *Babesia* parasites.

Here we define intra-erythrocytic *B*. *divergens* parasites directly after invasion as mono-nucleated rings (1N nuclear content), which then rapidly progress to metabolically active but still mono-nucleated, haploid trophozoite populations. These forms are only morphologically distinguishable based on anaplasmoidy in rings compared to the more rounded / ovoid trophozoites and not on differences in nuclear content; further specific classification would require metabolic flux data. With the associated nuclear content information provided in this paper, we were able to indicate the subsequent progression of mono-nucleated ring / trophozoites to bi-nucleated paired piriforms (2N nuclear content) during binary fission in which a single parasite undergoes a single nuclear division event resulting in two daughter merozoites. During this nuclear division event, the nucleus becomes typically V-shaped (as observed with fluorescent microscopy). The formation and origin of multi-nucleated tetrads is less clear. These parasites may undergo two nuclear division cycles that may result in the formation of four daughter merozoites. Binary fission dictates the duplication of nuclear content in a cell, followed by DNA segregation and finally cytokinesis. The formation of tetrads would therefore imply either that (a) a duplicate binary fission event occurred simultaneously from a single ring, visible as the characteristic cross morphology prior to cytokinesis or (b) that a minor proportion of paired piriforms would not undergo cytokinesis after DNA replication but rather undergo a second round of DNA replication, resulting in the tetrad formations with a 4N nuclear content prior to cytokinesis and ultimately to the formation of 4 daughter merozoites. High-resolution real-time microscopy evaluation is needed to address these possibilities. Since tetrad forms are infrequently observed in culture compared to the other morphological forms, tetrad formation is either relatively rare (only 10–25% of the total parasite population) or occurs rapidly (with quick kinetics of cytokinesis) such that these forms are rarely observed. With an optimal multiplication index of 2–3, it does not make a major contribution to *B*. *divergens* parasite proliferation *in vitro*. All merozoites of *B*. *divergens* parasites are typically piriform and joined by their pointed ends and also do not fill the complete erythrocyte, as expected of ‘small babesiae’. The kinetics of invasion (and re-invasion) after initial contact between the parasite and the erythrocyte, proceeds rapidly, between 45 seconds and 10 minutes [21].

**Fig 6:**
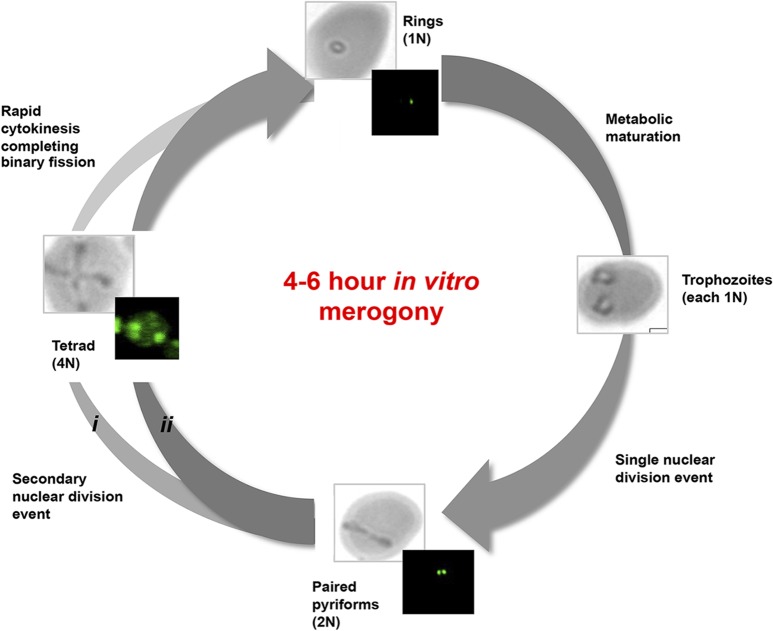
Model of the intra-erythrocytic developmental cycle of *B*. *divergens* parasites. Merozoites rapidly infect uninfected erythrocytes and develop into anaplasmoid ring stage parasites containing a haploid genome (single nuclei content, 1N). Following metabolic maturation in the feeding trophozoite parasites, DNA replication occurs through a single fission event resulting in bi-nucleated (2N) paired piriforms prior to cytokinesis. The formation of tetrads may occur either from (i) a duplicate binary fission event which occurs simultaneously from a single ring, visible as the characteristic cross morphology prior to cytokinesis or (ii) a minor proportion of paired piriforms do not undergo cytokinesis after DNA replication but rather undergo a second round of DNA replication, resulting in the tetrad formations with a 4N nuclear content prior to cytokinesis. Cytosol separation and membrane formation completes the cytokinesis and results in the production of 2–4 daughter merozoites, completing the merogony. In *in vitro B*. *divergens* cultures, the process is completed within 4 hours, resulting in significant increases in new ring parasite populations and concurrent increases in parasitemia, with a multiplication index of 2-fold.

We therefore hypothesize a parasitic propagation diagram based on the morphological observations, DNA measurements, temporal distribution and transcriptome expression dynamics (Fig 6). If an increase in parasitemia and associated increased ring populations is taken as indicators of new infections, then the significant increase in newly infected erythrocytes (ring formations) observed here during the first 4–6 hours in culture provides indications of the kinetics of the *B*. *divergens in vitro* IDC merogony. This is markedly quicker than previous reports where the *in vitro* life cycle of *B*. *divergens* parasites was claimed to last around 8 hours under the culture conditions used in that study [14]. However, the 2 hourly evaluation performed in our study enabled a finer analysis of the IDC progression kinetics that was not probed previously.

The asexual *Babesia* parasitic life cycle has only been described within the erythrocytes of their vertebrate hosts and the salivary glands of the tick vector, with limited data currently available concerning the *Babesia* sexual life cycle [22]. Only two studies have, however, reported on the visualization of intra-erythrocytic gametocytes in *B*. *divergens* [12,23]. Our temporal evaluation of *B*. *divergens* revealed contrasting results to that achieved with the optimized flow cytometry strategy during the interrogation of mixed infected erythrocyte populations. The overall population distribution (associated with temporal evaluation) remained predominantly paired piriforms followed by rings, tetrads and multiple infections. Comparatively, the overall population distribution observed with flow cytometry during the interrogation of mixed infected erythrocyte populations was predominantly ring formations followed by paired piriforms, tetrads and multiple infections. The inability to accurately visualize and characterize gametocytes with light microscopy within mixed *B*. *divergens* cultures may have contributed these contrasting results. However, we analyzed our transcriptional data set for genes characterizing gametocytes (i.e. *bdccp 1; bdccp 2* and *bdccp 3*) [24]), but these were not differentially affected in our data set. This can be interpreted as: 1) we did not observe gametocytes in *B*. *divergens* cultures *in vitro* (supporting our morphological descriptors characterising merozoites) or 2) the parasites are not under undue physiological stress, which have been implicated to result in increased gametocytogenesis. The transcriptional response that we do observe therefore mimics typical parasite physiology. Flow cytometry measurements may therefore include ring forms as well as gametocytes, thereby increasing the DNA content. Morphological findings suggest that a trend in parasitic development from one stage to the next can be observed and that all stages reach a plateau in their developmental process.

Transcriptome analyses of the initiate culture revealed an expression pattern for *B*. *divergens* parasites. The draft *B*. *divergens* genome (recently deciphered) in combination with the information presented here, can facilitate future *Babesia* related studies and improve our understanding of the parasites biology, host-parasite interaction as well as improve control and treatment strategies [25,26]. Additionally, the information presented here represents a preliminary catalog for *B*. *divergens* gene expression during its IDC, measured over time. The differential transcript abundance analysis identified several activated and repressed transcripts associated with parasitic growth and development, which may have contributed to changes in parasite population distributions. The transcriptional analysis was subsequently linked to the morphological findings. The initial stress response (between 0–4 hours) potentially induced by the addition of complete culture media, may have influenced the expression pattern. To minimize the possible stress response effects, an alternative expression pattern was suggested for the hierarchical clustered data set, which ranged between 4–16 hours. Functional annotation of the visualized transcripts and transcriptional response revealed predominantly activated and repressed components associated with transcription, translation, protein turnover, cellular and metabolic response as well as stress defense mechanisms, confirming the observed change in parasite population distributions (increases in ring and paired piriforms). The transcriptome mirrors the *Babesia* genome with glycolysis and components of the TCA cycle, glycerolipid and glycerophosphospholipid metabolism, pyrimidine and associated nucleotide synthesis, amino acid synthesis and certain components associated to apicoplast metabolism [11]. Moreover, almost a tenth of the differential transcriptome is associated with expression of transcripts in the *ves1* family (expressing VESA1). Similar to the malaria parasite, *Babesia* spp use antigenic variation to escape detection by the host’s immune system [26]. The hierarchical clustering employed here indicated that some of these biological processes do however show differential expression (e.g. protein expression and transport). Overall, the transcriptionally permissive state of the *B*. *divergens* IDC resembles that of the IDC transcriptome of *P*. *falciparum* parasites (with the exception of silenced virulence genes) [27]. In the latter, phase-ordering indicated a ‘just-in-time’ transcriptional activation, with transcripts finely associated with highly synchronized specific stages in the IDC. Even through the data presented here for *B*. *divergens* implies molecular control factors involved in the IDC, fine resolution temporal analysis of this parasite will be confounded as the population is composed of mixed stages, making stage-specific analysis difficult.

More comprehensive identifications of novel compounds against veterinary *Babesia* species have only recently gained attention [28]. However, for screening platforms to identify effective anti-babesiacidal compounds, several lessons may be learnt from highly advanced screening strategies from other thoroughly investigated diseases like malaria. As such, anti-malarial screening strategies have been clearly delineated and require early decision making based on target product and target candidate profiles. Particularly for *in vitro* hit identification, the ability of novel compounds to target specific (or all) stages of malaria parasite development as well as their speed of action is used to classify these *in vitro* specific profiles, thereby enabling their further prioritization [29,30]. Similar strategies are required for screening of anti-babesiacidal drugs, as evaluation of the stage-specific nature of compounds would be imperative in understanding it’s mode-of-action and examine new treatment and dosage strategies.

Current evaluation of growth-inhibiting effects of potential anti-babesiacidal compounds rely on either microscopic examination of Giemsa-stained smears and / or the evaluation of incorporation of isotopes into *in vitro Babesia* cultures [31], with neither of these allowing stage-specific or temporal evaluation of compound action. Light microscopy is time-consuming, subjective, labor intensive and operator dependent with poor quantitative robustness. Although isotopic techniques overcome the latter, this is being replaced by cost effective, reliable and non-radioactive fluorescence-based assays. Apart from the use of such dyes in plate-reader formats (enabling high-throughput screening platforms) [28] these assays are usually performed without evaluation of stage-specificity. However, the possibility to combine these dyes with techniques such as flow cytometry will allow for an additional level of characterization that has proved useful to address these caveats. One such example has been the successful application to the stage and temporal evaluation of anti-malarial compounds [17,32]. The expansion of our biological knowledge of *B*. *divergens* parasites’ intra-erythrocytic development through the molecular blueprint of its complete transcriptome provided in this paper should enhance future discovery of novel anti-babesiacidal drugs.

## Materials and Methods

### Ethics statement

Approval for the importation and *in vitro* cultivation of *B*. *divergens* was obtained from the South African Department of Agriculture, Forestry and Fisheries. Human blood and sera was collected from volunteer donors and used for cultivation with ethical approval (University of Pretoria Faculty of Natural and Agricultural Sciences Ethics Committee approved the project protocol with identification number EC120821-077). Volunteer donation was based on written informed consent from only adult donors at a registered phlebotomy facility. No minors were allowed to donate blood in this study. Parasite cell culturing was based on CDC criteria for such research. No animals were used in this study. All parasites were grown under *in vitro* conditions only.

### *In vitro* cultivation of intra-erythrocytic *B*. *divergens* and *P*. *falciparum* parasites

The Rouen 1987 strain of *B*. *divergens* was kindly provided by Dr. Stephane Delbecq (Laboratoire de Biologie Cellulaire et Moleculaire UFR Pharmacie, Montpellier, France). Asynchronous *B*. *divergens* parasite cultures were maintained in human erythrocytes (type O^+^) suspended in complete culture medium [RPMI-1640 medium supplemented with 25.2 mM HEPES, 22.2 mM D-glucose, 50 mg/l hypoxanthine, 21.4 mM sodium bicarbonate, 48 mg/l gentamycin (Sigma)] further supplemented with 10% human serum [33]. Cultures were maintained at 37°C in a gaseous environment of 90% N_2_, 5% O_2_ and 5% CO_2_ on a rotary platform (60 rpm) at ~5% hematocrit and 10–15% parasitemia, with daily media replacement. Comparatively, intra-erythrocytic *P*. *falciparum* (3D7 strain) parasites were obtained from the MR4 (www.mr4.org) and were maintained at a 5% hematocrit, 2–5% parasitemia in complete culture medium [RPMI-1640 medium supplemented with 25.2 mM HEPES, 22.2 mM D-glucose, 50 mg/l hypoxanthine, 21.4 mM sodium bicarbonate, 48 mg/l gentamycin (Sigma)] further supplemented with 0.5% Albumax II (Invitrogen) [34] in the same gaseous environment under shaking conditions as above.

### Microscopy

For both intra-erythrocytic *B*. *divergens* and *P*. *falciparum* parasites, Giemsa-stained thin smears light microscopy was used for the daily determination of both the parasitemia and morphology. Thin blood smears were prepared, air dried, fixed with methanol and stained for 5 minutes prior to examination. The percentage parasitemia was determined as the number of infected erythrocytes per 100 cells, with a minimum of 1000 erythrocytes counted [35]. Asynchronous intra-erythrocytic *B*. *divergens* parasites (5% hematocrit, 10–15% parasitemia) were examined every two hours over a 16-hour time period using Giemsa-stained smears and light microscopy. The asexual developmental life stages were classified as rings, paired piriform and tetrad and/or multiple infection formations. Fluorescent microscopy (Zeiss Axiovert 200 fluorescent microscope) was also used to examine intra-erythrocytic *B*. *divergens* parasites using the Axiovision release 4.8.2 software for analyses.

### Flow cytometric analysis using SYBR Green I fluorescence

#### Determining parasitemia

The parasitemia of both intra-erythrocytic *B*. *divergens* and *P*. *falciparum* parasites was measured using flow cytometry following the staining of nucleic acids with SYBR Green I (Invitrogen). Uninfected erythrocytes (negative control; 5% hematocrit), asynchronous *P*. *falciparum* samples (positive control; 5% hematocrit, 2% parasitemia) and asynchronous *B*. *divergens* samples (5% hematocrit, 12% parasitemia) were used either unfixed or fixed with 1 ml of 0.025% glutaraldehyde for 45 min and kept at 4°C until use. Glutaraldehyde fixed cells was washed twice with phosphate buffered saline (1x PBS: 137 mM NaCl, 2.7 mM KCl, 1.8 mM KH_2_PO_4_, 10 mM Na_2_HPO_4_.7H_2_O, pH 7.4) and re-suspended in a final volume of 50 μl PBS. *P*. *falciparum* parasites were stained at room temperature with 20 μl 1:1000 SYBR Green I (10 000x SYBR Green I):PBS solution for 30 minutes in the dark [36]. Glutaraldehyde fixed *B*. *divergens* cells were stained with various dilutions of SYBR Green I (10 000x SYBR Green I):PBS to determine optimal fluorescence. Both unfixed and fixed *B*. *divergens* parasites were stained with 20 μl 1:100 and 1:1000 SYBR Green I (10 000x SYBR Green I):PBS solutions respectively and incubated at 37°C for 30 minutes in the dark.

In all cases, SYBR Green I fluorescence was measured using the BD FACS Aria I flow cytometer with fluorescence emission collected at an excitation wavelength of 488 nm, 502 nm long-band-pass and 530 nm band-pass emission filter equipped with a 488-nm, 633-nm, 405-nm and 375-nm near UV laser. SYBR Green I fluorescent dye was detected with the FITC (515–545 nm) band pass filter. The number of events recorded were gated for 10 000 events outside of the erythrocyte’s background signal, thereby recording and analyzing approximately 10^4^ infected erythrocytes per sample. The gating strategy for quantification of *B*. *divergens* was plotted on forward (FSC) versus side (SSC) scatter density plots and used to gate and sort cell populations.

Intra-erythrocytic *B*. *divergens* parasitemia was calculated based on the number of erythrocytes counted (in relation to the relative fluorescence intensities) and displayed on single parameter histogram plots. Parasitemia validation was done on all samples with light microscopy using Giemsa-stained smears. Three independent experiments were conducted in triplicate with analysis and compensation of data performed by FlowJo version 9.1 (Tree Star). Statistical data analysis was performed with GraphPad Instat (version 6.04). In all cases, the percentage parasitemia associated with the x-axis was plotted against the units fluorescence (RFU) associated with the y-axis and subsequently analyzed by linear regression to determine the goodness of fit (R^2^-value).

#### Determining stage distribution

DNA replication and nuclear division of intra-erythrocytic *B*. *divergens* parasites were determined using SYBR Green I fluorescence and correlated morphologically to the different developmental stages of the parasite. Intra-erythrocytic *B*. *divergens* cultures (5% hematocrit, 12% parasitemia, 50 μl, unfixed) were processed as described above and stained with 20 μl 1:100 SYBR Green I (10 000x SYBR Green I):1xPBS solution and incubated at 37°C for 30 minutes in the dark. Fluorescence was measured as described above. Cell sorting was subsequently set up for a three-way sort of populations P1, P2, P3 and P4 to at least 1 million cells per individual collection tube. Following cell sorting, individual populations were subjected to light microscopy. The content of each collection tube was centrifuged for 10 minutes at 8000x*g* to obtain a cell pellet which was dissolved in 20 μl 1xPBS solution, placed on a glass slide and examined with light microscopy.

### Transcriptome analysis

A custom designed DNA microarray slide was used for gene expression analysis. The base composition probe design strategy and probe selection parameters were selected according to Agilent Technologies eArray 60-mer platform specifications. Available *B*. *bovis* sequences were downloaded and retrieved from the National Center for Biotechnology Information (NCBI) (http://www.ncbi.nlm.nih.gov/) and supplemented with additional published sequence data [5]. Selected probes were randomly distributed across the array using the 8x 15K design format. All arrays were ordered from Agilent Technologies (https://earray.chem.agilent.com/earray/).

#### RNA isolation, DNA microarray hybridizations and quality control

Total RNA was isolated from three biological replicate samples (5% hematocrit, 12% parasitemia, 10 ml) collected at four time-points (0, 4, 9 and 16 hours) from newly initiated cultures and washed three times with 15 ml 1xPBS solution under RNAse-free conditions. TRI-Reagent fractionation was followed by clean-up using the Qiagen RNeasy Protect Mini kit (Qiagen). Contaminating genomic DNA was removed with a DNase I treatment (Qiagen). RNA integrity and quality was subsequently analyzed using the Experion automated electrophoresis system (Bio-Rad) at the ACGT Microarray Facility (University of Pretoria).

For gene expression profiling, a reference pool design strategy was followed which consisted of equal RNA quantities (4 μg), prepared from all three biological replicates collected over the 16 hour time period. First-strand cDNA synthesis was performed by incubating 4 μg RNA with 250 pmol oligo(dT_25_) and 775 pmol random primer 9 for 10 minutes at 70°C, followed by cooling for 10 minutes on ice. Reverse transcription and aminoallyl-dUTP (5-(3-aminoallyl)-2’-deoxyuridine-5’ triphosphate) incorporation were performed simultaneously [27], with minor modifications in reaction time [37] using 340 units Superscript III (Invitrogen). Contaminating RNA template was removed by RNA hydrolysis of the cDNA template with 0.5 M EDTA and 1 M NaOH for 15 minutes at 65°C before samples were purified with a NucleoSpin Extract II PCR Clean-up kit (Macherey-Nagel). Purified cDNA samples (1.5 μg) were dried *in vacuo* and resuspended in 2.5 μl RNAse-free water. Both Cy3 (reference pool) and Cy5 (samples) fluorescent dyes (GE Healthcare Life Sciences) were dissolved in DMSO and coupled to the cDNA samples at pH 9. Excess (unincorporated) dye was removed using the QIAquick PCR Purification Kit (Qiagen). Overnight hybridization at 65°C (rotation speed of 10), washing and post-processing were performed as described [27] at the ACGT Microarray Facility (University of Pretoria). Prior to slide scanning with the Axon GenePix 4000B scanner (Molecular Devices) slides were removed, washed and dried by centrifugation.

#### Microarray data analysis

Axon GenePix Pro 6.0 software (Molecular Devices) measured, recorded and analyzed images based on the software’s default settings as well as visual inspection of spots (adjusting the grid and flagging of low quality spots). Flagged features were ignored in subsequent analyses and given a zero weight value. Initial data analysis was performed using CLUSTER and TREEVIEW software [38]. Data was log transformed, normalized and mean centered prior to hierarchical clustering and subsequently ordered according to expression values (*M*-values) identified over time. Pearson correlation coefficients (r) were calculated within the R-statistical environment to identify expression similarities between each of the time points. The linear model for microarray data analysis (LIMMA) within the R-statistical environment (http://cran.r-project.org/) was used to simultaneously assess differential expression between several transcripts, collected at different time points. Adaptive background correction (offset = 50) was followed by within-array normalization (global LOWESS) and between-array normalization (Gquantile). Fold change was determined between all transcripts collected at different time-points using the empirical Bayesian statistics and subsequently expressed as *P*-values (corrected for false discovery rate). Transcripts were regarded as differentially expressed if a greater than 1.7 fold change (0.75 ≤ log_2_ratio ≤ -0.75) in either direction with *P*-value <0.05 were observed. A selected subset of differentially affected transcripts was constructed based on data filtering, fold change and statistical significance (log_2_FC, *P-*values, adjusted *P-*values and *t-*statistics). Functional annotation of transcripts was conducted using the desktop cDNA Annotation System software (dCAS) [18]. Large-scale BLAST searches were conducted with the gene ontology protein sequence database (GO) and eukaryotic orthologous group database (COG). Functional annotation were obtained based the piroplasmaDB annotations (www.piroplasmaDB.org) and verified by Uniprot (http://www.uniprot.org/uniprot/) and BLAST2GO (B2G) [39].

## Supporting Information

S1 Text Explanation of the development of a fluorescent cell biological evaluation technique to detect intra-erythrocytic *B*. *divergens* parasites. (DOCX)

S1 Fig Flow cytometric analysis of intra-erythrocytic *B*. *divergens* parasites. Uninfected erythrocytes were analysed in parallel to *B*. *divergens* infected erythrocytes, either fixed (0.025% glutaraldehyde for 45 min) or unfixed. Cells were subsequently stained with either 1:100 and 1:1000 SYBR Green I (30 min, dark, room temperature). (A) Dotblot analysis and (B) histograms of 1) uninfected, unfixed, stained erythrocytes; 2–5) *B*. *divergens* infected erythrocytes either unfixed (2 & 3) or fixed (4 & 5). In panels 2 and 4 cells were stained with 1:100 SYBR Green I and in panels 3 and 5 with 1:1000 SYBR Green I. Erythrocytes infected with *P*. *falciparum* parasites were comparatively analysed in panel 6 (glutaraldehyde fixed and stained with 1:1000 SYBR Green I. (C) Effect of SYBR Green I concentrations on parasitemia determined from both unfixed and fixed *B*. *divergens* infected erythrocytes. Results are the mean of three independent experiments each performed in triplicate (± S.E.). Significance is indicated at *P*<0.001 (*) (unpaired Student-t test). (D) Linear correlation analysis (R^2^ value of 0.98) of *B*. *divergens* parasitemia detection between light microscopy and flow cytometry. Data are the mean of three independent experiments each performed in triplicate (± S.E.). Confidence levels (95%) indicated by dashed lines.
(DOCX)

S1 Table Differentially affected transcripts identified in the initiate culture before culture adaptation. (DOCX)
