# Supplementary material for: Morphological and Molecular Descriptors of the Developmental Cycle of Babesia divergens Parasites in Human Erythrocytes
Source: PLoS Negl Trop Dis. 2015 May 8;9(5):e0003711. doi: 10.1371/journal.pntd.0003711 (PMC4425553; doi:10.1371/journal.pntd.0003711)
Supplement: S1 Text — (DOCX) [file pntd.0003711.s001.docx]

**Development of a fluorescent cell biological evaluation technique to detect intra-erythrocytic *B. divergens* parasites**

Flow cytometry (using SYBR Green I fluorescence as nuclear marker) sensitively and accurately discriminated *Babesia* infected erythrocytes from their uninfected partners (Fig 1A). A 1:100 SYBR Green I (10 000x SYBR Green I):PBS solution was able to discriminate uninfected from three separate infected erythrocyte populations with the highest resolution (Fig 1A_2_; A_3_). However, the use of glutaraldehyde as fixative at both SYBR Green I concentrations, resulted in a marked decrease in resolution compared to the unfixed samples (Fig 1A_4_; A_5_ and Fig 1C). Asynchronous, unfixed, stained *P. falciparum* was used as positive control as distinct populations have been validated previously using a similar approach (Fig 1A_6_)[[17](#_ENREF_17),[18](#_ENREF_18)].

The optimal staining procedure (1:100 SYBR Green I solution with unfixed parasites) was also optimal to accurately determine the percentage infected erythrocytes (parasitemia) of *B. divergens* cultures (Fig 1B). Even though a 1:100 concentration of the fluorescent dye was more informative with regards to population resolution, parasitemia could still be accurately detected with a 10-fold dilution of SYBR Green I from 1:100 to 1:1000 (Fig 1B_1;_ B_2_). Fixation of *B. divergens* parasites detrimentally affected the ability to determine parasitemia accurately by flow cytometry, resulting in a significant 2-fold reduction in reported parasitemia under these conditions (Fig 1B_4_; B_5_ and Fig 1C).

The flow cytometric assay was subsequently verified as alternative method of determining parasitic proliferation compared to light microscopy, by analyzing asynchronous *B. divergens* samples in triplicate with both light microscopy and flow cytometry. A strong linear correlation (R^2^ = 0.98) was observed between the microscopic and flow cytometric analyses (unfixed cells, 1:100 SYBR Green I dilution) based on the parasitemia obtained either with light microscopy or determined with the BD FACS Aria I flow cytometer (Fig 1D).
