# Supplementary figures and images for: Morphological and Molecular Descriptors of the Developmental Cycle of Babesia divergens Parasites in Human Erythrocytes
Source: PLoS Negl Trop Dis. 2015 May 8;9(5):e0003711. doi: 10.1371/journal.pntd.0003711 (PMC4425553; doi:10.1371/journal.pntd.0003711)

**
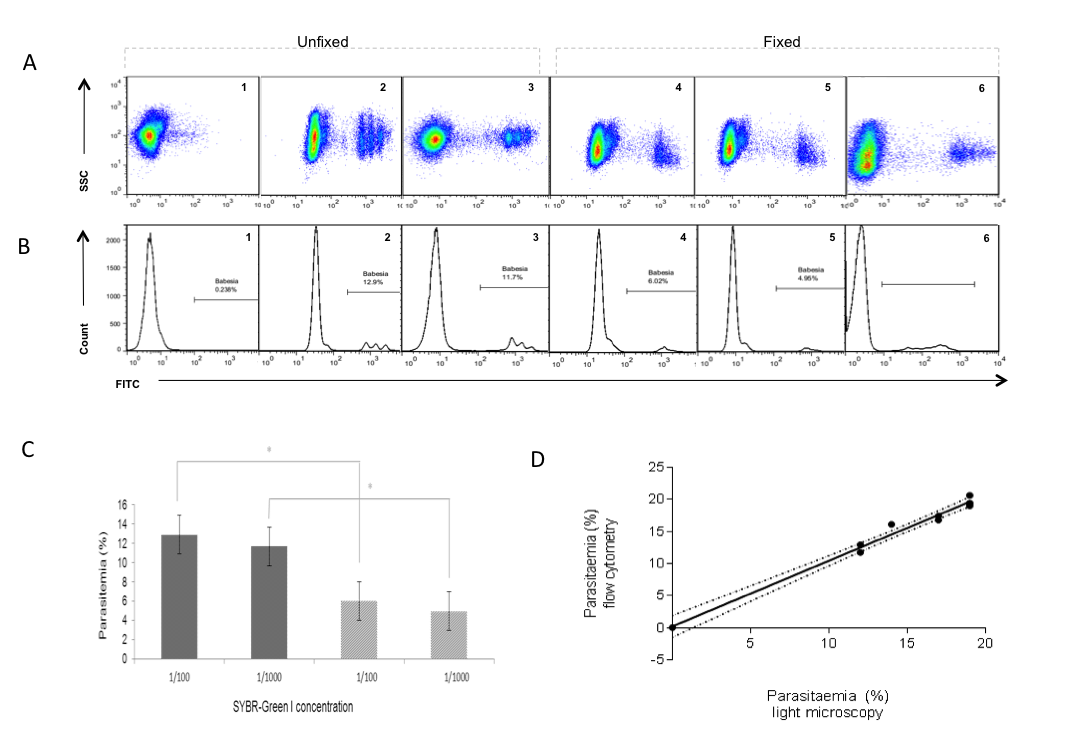
**

Supplement: S1 Fig — Uninfected erythrocytes were analysed in parallel to B. divergens infected erythrocytes, either fixed (0.025% glutaraldehyde for 45 min) or unfixed. Cells were subsequently stained with either 1:100 and 1:1000 SYBR Green I (30 min, dark, room temperature). (A) Dotblot analysis and (B) histograms of 1) uninfected, unfixed, stained erythrocytes; 2–5) B. divergens infected erythrocytes either unfixed (2 & 3) or fixed (4 & 5). In panels 2 and 4 cells were stained with 1:100 SYBR Green I and in panels 3 and 5 with 1:1000 SYBR Green I. Erythrocytes infected with P. falciparum parasites were comparatively analysed in panel 6 (glutaraldehyde fixed and stained with 1:1000 SYBR Green I. (C) Effect of SYBR Green I concentrations on parasitemia determined from both unfixed and fixed B. divergens infected erythrocytes. Results are the mean of three independent experiments each performed in triplicate (± S.E.). Significance is indicated at P<0.001 (*) (unpaired Student-t test). (D) Linear correlation analysis (R2 value of 0.98) of B. divergens parasitemia detection between light microscopy and flow cytometry. Data are the mean of three independent experiments each performed in triplicate (± S.E.). Confidence levels (95%) indicated by dashed lines. (DOCX) [file pntd.0003711.s002.docx]
