# Supplementary material for: Morphological and Molecular Descriptors of the Developmental Cycle of Babesia divergens Parasites in Human Erythrocytes
Source: PLoS Negl Trop Dis. 2015 May 8;9(5):e0003711. doi: 10.1371/journal.pntd.0003711 (PMC4425553; doi:10.1371/journal.pntd.0003711)
Supplement: S1 Table — (DOCX) [file pntd.0003711.s003.docx]

**S1 Table. Differentially affected transcripts identified in the initiate culture before culture adaptation.**

| **Transcript ID (PiroplasmaDB)** | **Annotation** | **logFC** | ***E-*value** |
| --- | --- | --- | --- |
| BBOV_III000960 | RNA polymerase III | -2.02 | 1E-58 |
| BBOV_IV005830 | F0F1-type ATP synthase | 2.06 | 2E-47 |
| BBOV_IV007210 | Succinate dehydrogenase | 2.92 | 3E-38 |
| BBOV_IV001690 | Aspartyl-tRNA synthetase | 1.46 | 8E-25 |
| BBOV_II005700 | Ca2+ transporting ATPase | -2.98 | 8E-23 |
| BBOV_IV007190 | Dihydrolipoamide dehydrogenase | 2 | 9E-21 |
| BBOV_IV010920 | GTPase Ran/TC4/GSP1 | 0.96 | 1E-18 |
| BBOV_I001930 | Putative arsenite-translocating ATPase | -1.74 | 6E-18 |
| BBOV_IV001010 | Fe-S oxidoreductase | 1.44 | 9E-18 |
| BBOV_II006920 | Citrate synthase | -2.55 | 8E-17 |
| BBOV_III006690 | Vesicle coat complex AP-1/AP-2/AP-4 | -2.22 | 1E-16 |
| BBOV_II002690 | Global transcriptional regulator | -2.34 | 2E-15 |
| BBOV_II005600 | mRNA splicing factor | 1.59 | 2E-15 |
| BBOV_III000190 | ATP-dependent RNA helicase | -4.37 | 3E-15 |
| BBOV_IV004470 | U4/U6-associated splicing factor PRP4 | -1.58 | 3E-15 |
| BBOV_III008650 | Putative methionine aminopeptidase | 1.6 | 3E-15 |
| BBOV_IV000850 | Protein containing a U1-type Zn-finger | 2.07 | 3E-15 |
| BBOV_II004950 | Adenylosuccinate synthase | -2.13 | 1E-13 |
| BBOV_IV006160 | Mitochondrial polypeptide chain release factor | -2.42 | 2E-12 |
| BBOV_II002330 | 20S proteasome, regulatory subunit | 1.58 | 3E-12 |
| BBOV_III007370 | Serine/threonine protein kinase | -2.6 | 5E-11 |
| BBOV_IV003180 | Predicted transporter | 1.33 | 1E-10 |
| BBOV_III000400 | Para-hydroxybenzoate-polyprenyl transferase | 1.3 | 3E-10 |
| BBOV_III009930 | Ubiquinol cytochrome c reductase | -1.83 | 0.000000002 |
| BBOV_IV000740 | Vesicle coat complex COPII | 1.2 | 0.000000002 |
| BBOV_III000980 | 60S ribosomal protein L7 | 1.05 | 0.000000003 |
| BBOV_I002600 | RNA polymerase II | 2.338 | 0.000000008 |
| BBOV_IV008310 | Ethanolamine kinase | -2.34 | 0.00000001 |
| BBOV_IV007450 | Mitochondrial/chloroplast ribosomal protein L2 | 2.098 | 0.00000001 |
| BBOV_IV004330 | Serine protease | -1.68 | 0.00000002 |
| BBOV_IV001730 | Ubiquitin carboxyl-terminal hydrolase | -2.43 | 0.00000003 |
| BBOV_II007270 | Predicted small molecule transporter | 1.03 | 0.0000001 |
| BBOV_IV004490 | Chaperone-dependent E3 ubiquitin protein ligase | 1.097 | 0.0000001 |
| BBOV_II002960 | Vesicle coat complex COPI, gamma subunit | -1.54 | 0.0000002 |
| BBOV_I004230 | Small nuclear ribonucleoprotein (snRNP) | 1.121 | 0.0000002 |
| BBOV_II006560 | Molecular chaperone (DnaJ superfamily) | 2.04 | 0.0000002 |
| BBOV_IV005570 | Metallopeptidase | -2.56 | 0.0000003 |
| BBOV_III008970 | Membrane coat complex Retromer, subunit VPS29/PEP11 | -3.01 | 0.000004 |
| BBOV_I000180 | CCAAT-binding factor, subunit A (HAP3) | -1.83 | 0.000004 |
| BBOV_IV006920 | mRNA splicing factor | 2.007 | 0.000006 |
| BBOV_IV000490 | Acyl-CoA-binding protein | -2.36 | 0.00001 |
| BBOV_IV001200 | ATP-dependent RNA helicase | -2.17 | 0.00001 |
| BBOV_III011610 | RNA pseudouridylate synthases | 2.116 | 0.00001 |
| BBOV_IV006860 | SCF ubiquitin ligase | -4.35 | 0.00005 |
| BBOV_III008510 | Spliceosomal protein FBP21 | -2.34 | 0.00006 |
| BBOV_I001880 | Vesicle coat complex AP-2 | -1.6 | 0.00006 |
| BBOV_III009670 | Dihydroorotase | -2.14 | 0.0001 |
| BBOV_II005540 | Cysteine protease required for autophagy - Apg4p | -1.67 | 0.0002 |
| BBOV_III005690 | Uncharacterized mRNA-associated protein RAP55 | 2.252 | 0.0002 |
| BBOV_II000110 | Wiskott Aldrich syndrome proteins | -2.61 | 0.0003 |
| BBOV_III004170 | Exosomal 3'-5' exoribonuclease complex subunit Rrp40 | 1.712 | 0.0003 |
| BBOV_I001560 | Protein kinase PITSLRE and related kinases | -1.2 | 0.0004 |
| BBOV_IV004500 | Predicted GTP-binding protein | -2.3 | 0.0006 |
| BBOV_IV004600 | Uncharacterized conserved protein | 2.257 | 0.0006 |
| BBOV_II005170 | Permease of the major facilitator superfamily | 1.974 | 0.0009 |
| BBOV_II005420 | Predicted seven transmembrane receptor | -1.77 | 0.001 |
| BBOV_II007370 | Methyltransferases | 2.839 | 0.001 |
| BBOV_II002840 | RNA polymerase II assessory factor Cdc73p | -1.71 | 0.002 |
| BBOV_II003490 | Molecular chaperone (DnaJ superfamily) | -2.69 | 0.003 |
| BBOV_IV011250 | N-terminal acetyltransferase | 2.291 | 0.003 |
| BBOV_IV001370 | U4/U6-associated splicing factor PRP4 | 2.456 | 0.003 |
| BBOV_II004880 | Zn-finger transcription factor | -2.23 | 0.004 |
| BBOV_II002070 | Predicted membrane protein | 3.031 | 0.004 |
| BBOV_III008270 | DEAD box protein | 1.778 | 0.005 |
| BBOV_IV010050 | Amine oxidase | -1.99 | 0.007 |
| BBOV_III004010 | HAT (Half-A-TPR) repeat-containing protein | -3.29 | 0.011 |
| BBOV_III003570 | 60s ribosomal protein L24 | 1.648 | 0.018 |
| BBOV_IV005380 | Predicted membrane proteins | -1.86 | 0.019 |
| BBOV_IV003000 | Glycine dehydrogenase (decarboxylating) | 1.455 | 0.02 |
| BBOV_IV003070 | Junctional membrane complex protein Junctophilin | 1.916 | 0.022 |
| BBOV_II005550 | Ultrahigh sulfur keratin-associated protein | -2.49 | 0.023 |
| BBOV_IV005630 | Beta-1,3-glucuronyltransferase B3GAT1 | -3.14 | 0.031 |
| BBOV_II006720 | Zn-finger protein | -1.62 | 0.031 |
| BBOV_II003780 | Predicted spermine/spermidine synthase | -2.1 | 0.032 |
| BBOV_I004510 | Proteins containing Ca2+-binding EGF-like domains | -1.78 | 0.032 |
| BBOV_II006330 | Nucleotide excision repair factor NEF2 | 2.383 | 0.038 |
| BBOV_IV000220 | Tuberin - Rap/ran-GTPase-activating protein | -2.52 | 0.039 |
| BBOV_II004720 | G protein-coupled seven transmembrane receptor | 2.86 | 0.041 |
| BBOV_II001290 | Gamma-tubulin complex | 1.339 | 0.042 |
| BBOV_II002810 | SNARE protein TLG2 | -2.93 | 0.043 |
| BBOV_IV000280 | KRR1-interacting protein involved in 40S ribosome biogenesis | 2.431 | 0.044 |
| BBOV_III005640 | Uncharacterized conserved protein | 1.731 | 0.046 |
| BBOV_III010170 | Lipid exporter ABCA1 and related proteins, ABC superfamily | -1.53 | 0.047 |
| BBOV_IV006000 | Nuclear division RFT1 protein | 1.374 | 0.054 |
| BBOV_III004410 | Extracellular matrix glycoprotein Laminin | -2.23 | 0.057 |
| BBOV_II007240 | Predicted transcription factor DATF1 | 2.365 | 0.064 |
| BBOV_I004150 | ATP synthase | 1.916 | 0.071 |
| BBOV_IV009810 | tRNA(1-methyladenosine) methyltransferase | -5.25 | 0.074 |
| BBOV_II003240 | Long chain fatty acid acyl-CoA ligase | -2.26 | 0.079 |
| BBOV_I003670 | Predicted integral membrane protein | 2.382 | 0.081 |
| BBOV_I001610 | Amino acid transporters | -2.09 | 0.087 |
| BBOV_IV006740 | ATP binding protein | -2.89 | 0.09 |
| BBOV_IV000010 | UDP-N-acetylglucosamine pyrophosphorylase | 1.397 | 0.092 |
| BBOV_IV005420 | WD40 repeat-containing protein L2DTL | 1.835 | 0.094 |
| BBOV_III005300 | Uncharacterized conserved protein | -2.48 | 0.099 |
| BBOV_IV000890 | Vesicle coat protein clathrin | -2.06 | 0.1 |
| BBOV_IV001240 | Mitochondrial import inner membrane translocase | -3.7 | 0.11 |
| BBOV_II001510 | Predicted mitochondrial cholesterol transporter | -2.74 | 0.11 |
| BBOV_IV011450 | Adenylate/guanylate cyclase | -2.59 | 0.11 |
| BBOV_IV005880 | Aryl-hydrocarbon receptor | -1.59 | 0.11 |
| BBOV_IV007920 | RNA polymerase II | 1.924 | 0.11 |
| BBOV_III008840 | Ammonia permease | 2.098 | 0.11 |
| BBOV_II006130 | Inositol 1,4,5-trisphosphate receptor | -3.15 | 0.14 |
| BBOV_IV006800 | Synaptic vesicle transporter SV2 (major facilitator superfamily) | 1.601 | 0.14 |
| BBOV_IV010220 | Pleiotropic drug resistance proteins (PDR1-15), ABC superfamily | -2.5 | 0.16 |
| BBOV_III001630 | Inhibitor of type V adenylyl cyclases | -1.79 | 0.16 |
| BBOV_III009610 | Protein geranylgeranyltransferase type II | -1.33 | 0.17 |
| BBOV_II004030 | Tuberin - Rap/ran-GTPase-activating protein | 1.657 | 0.17 |
| BBOV_I003900 | Uncharacterized conserved protein | 2.178 | 0.19 |
| BBOV_III009840 | Putative trehalase | -2.33 | 0.2 |
| BBOV_I003970 | Mitochondrial translation initiation factor 2 (IF-2; GTPase) | -2.11 | 0.2 |
| BBOV_I004860 | Predicted haloacid dehalogenase-like hydrolase | 1.831 | 0.2 |
| BBOV_II000880 | Uncharacterized conserved protein | 2.497 | 0.22 |
| BBOV_III000230 | GTPase-activating proteins | -2.89 | 0.23 |
| BBOV_II006680 | Transporter, ABC superfamily | 1.474 | 0.23 |
| BBOV_I003960 | Uncharacterized conserved protein | 1.682 | 0.23 |
| BBOV_III003710 | Vesicle coat complex COPII | 2.123 | 0.23 |
| BBOV_IV003280 | Nucleolar RNA-associated protein | 2.447 | 0.26 |
| BBOV_I004280 | Permease of the major facilitator superfamily | -1.67 | 0.28 |
| BBOV_II002370 | Anaphase promoting complex | 1.123 | 0.28 |
| BBOV_III001200 | Ras GTPase activating protein RasGAP/neurofibromin | 2.058 | 0.29 |
| BBOV_IV012010 | Smoothened and related G-protein-coupled receptors | 1.796 | 0.3 |
| BBOV_I001130 | Acetyl-CoA transporter | 2.153 | 0.3 |
| BBOV_IV004310 | Cytochrome c oxidase, subunit II | 1.398 | 0.34 |
| BBOV_IV007520 | Integrin beta subunit | 1.838 | 0.36 |
| BBOV_II007830 | Uncharacterized conserved protein | -3.36 | 0.38 |
| BBOV_III005710 | PAB-dependent poly(A) ribonuclease | 1.897 | 0.38 |
| BBOV_II006370 | SAM-dependent methyltransferases | -1.33 | 0.39 |
| BBOV_II003830 | N-acetylglucosaminyltransferase complex | 2.302 | 0.39 |
| BBOV_I001120 | Myosin class II heavy chain | 1.719 | 0.4 |
| BBOV_III008740 | Permease of the major facilitator superfamily | -2.42 | 0.43 |
| BBOV_I004990 | AAA+-type ATPase | 2.508 | 0.43 |
| BBOV_IV000290 | Sphingolipid hydroxylase | 2.851 | 0.46 |
| BBOV_III000620 | Peroxisomal NUDIX hydrolase | -1.51 | 0.5 |
| BBOV_V000330 | Pleiotropic drug resistance proteins (PDR1-15) | -2.98 | 0.51 |
| BBOV_III003550 | Predicted GTPase | 1.79 | 0.51 |
| BBOV_IV005430 | Signaling protein van gogh | -2.75 | 0.54 |
| BBOV_III005460 | Ultrahigh sulfur keratin-associated protein | -2.68 | 0.63 |
| BBOV_III011930 | Cell division control protein | 2.106 | 0.63 |
| BBOV_IV005210 | Ca2+ release channel | 1.687 | 0.65 |
| BBOV_I003990 | Predicted membrane protein | 2.049 | 0.66 |
| BBOV_III006710 | Spondins, extracellular matrix proteins | 2.637 | 0.73 |
| BBOV_IV000270 | RNA-binding protein | 1.515 | 0.75 |
| BBOV_IV000330 | Conserved developmentally regulated protein | -4 | 0.77 |
| BBOV_IV000900 | Glycoprotein hormone receptor | -2.32 | 0.78 |
| BBOV_II004340 | Mitochondrial glycerol-3-phosphate acyltransferase GPAT | -1.6 | 0.81 |
| BBOV_II006630 | Predicted membrane protein | 1.552 | 0.81 |
| BBOV_I003260 | Uncharacterized conserved protein | -2.9 | 0.9 |
| BBOV_I002830 | Ca2+-activated K+ channel | -1.73 | 0.91 |
| BBOV_III003240 | Ca2+ release channel | -1.68 | 0.93 |
| BBOV_II005500 | Para-hydroxybenzoate-polyprenyl transferase | 2.597 | 0.96 |
| BBOV_III000600 | Neural cell adhesion molecule L1 | 1.49 | 1.1 |
| BBOV_II001570 | Predicted G-alpha GTPase interaction protein | 3.03 | 1.1 |
| BBOV_IV004810 | Density-regulated protein related to translation initiation factor 1 (eIF-1/SUI1) | 1.548 | 1.2 |
| BBOV_II006950 | Rhomboid family proteins | -3.16 | 1.3 |
| BBOV_III000210 | Predicted membrane proteins | -2.48 | 1.3 |
| BBOV_I003550 | Signal recognition particle receptor | -2.28 | 1.3 |
| BBOV_II003330 | Sister chromatid cohesion protein SCC2 | -2.07 | 1.3 |
| BBOV_IV003580 | Glucosamine 6-phosphate synthetases | 1.906 | 1.6 |
| BBOV_IV010090 | Histone H3 (Lys4) methyltransferase complex | 2.045 | 1.6 |
| BBOV_IV010190 | 50S ribosomal protein L1 | 2.157 | 1.8 |
| BBOV_II001740 | Phosphatidic acid-preferring phospholipase A1 | 2.277 | 2.3 |
| BBOV_IV004070 | 2-oxoglutarate dehydrogenase | -1.37 | 2.5 |
| BBOV_III003960 | ATP-dependent RNA helicase A | -3.73 | 3 |
